# Development of an improved microneutralization assay for respiratory syncytial virus by automated plaque counting using imaging analysis

**DOI:** 10.1186/1743-422X-2-84

**Published:** 2005-11-09

**Authors:** Edyta Zielinska, Daiqing Liu, Hong-Yin Wu, Jorge Quiroz, Ruth Rappaport, Da-Ping Yang

**Affiliations:** 1 Clinical Immunology and Virology, Wyeth Vaccines Research, Pearl River, NY. USA

## Abstract

**Background:**

Respiratory syncytial virus (RSV) is the major cause of lower respiratory tract infection in infants and young children. Although several experimental RSV vaccines are under investigation, immuno therapy is the only treatment currently available. In assessing the immunogenicity of various vaccine formulations, a plaque reduction neutralization assay for the evaluation of RSV neutralizing antibody has been widely used. The method produces reliable results, but it is tedious and labor intensive as it relies on manual counting by laboratory personnel. To facilitate evaluation of phase II and phase III vaccine clinical trials, a more rapid, reliable and efficient neutralization assay is needed.

**Results:**

An improved microneutralization assay for quantifying RSV neutralizing antibodies was developed using an ImmunoSpot^® ^Series I Analyzer (Cellular Technology Ltd., Cleveland, OH) for automated plaque counting. The method is an improvement of the established classical microneutralization assay in which immunostained plaques on transparent tissue culture plates are counted manually under a dissecting microscope. Image analyzer technology allows for fully automated counting of plaques distributed throughout an entire well. Adjustments, such as the use of opaque tissue culture plates and the TMB substrate, True Blue™ (KPL, Gaithersburg, MD), were required to adapt the assay for optimal detection of plaques by the image analyzer. The suitability and the accuracy of the method for counting RSV plaques were determined by comparative testing of a reference serum and two control sera by manual and automated counting methods. The results showed that the two methods were highly correlated (R = 0.9580) and the titers generated by them were within two-fold.

**Conclusion:**

Our results demonstrate that the semi-automated assay is rapid and reliable. It provides results within two fold to the classical plaque microneutralization assay and is readily applied to the evaluation of neutralizing antibody titers in sera obtained from epidemiology or vaccine clinical trials.

## Background

Respiratory syncytial virus (RSV) is the most common form of lower respiratory viral infection affecting infants, the elderly, and immunocompromised individuals [1]. In severe cases, it may cause complications ranging from pneumonia and bronchiolitis to death [2]. While the most severe outcomes arise in patients with weakened or underdeveloped immune systems, RSV is also gaining notoriety as an important player in annual respiratory disease epidemics among healthy adults [3]. Consequently, there is an obvious unmet need for an efficacious vaccine. The development of a vaccine will require intensive evaluation of the immune response, which can be expedited by utilizing automation.

The neutralization assay is one of the most trusted and widely used methods employed for the detection of virus-specific neutralizing antibodies [4]. The power of the neutralization assay lies in its ability to detect biologically active antibodies. While there are many methods that provide information about different aspects of the immune response (e.g. cellular immunity, genetic markers, etc.) the neutralization assay remains a proven indicator of serological immunity for many viruses. In practice, however, the plaque neutralization assay is a laborious and time-consuming procedure, making it less suitable for testing the large numbers of samples that are obtained in clinical trials. Here, we demonstrate the utility of a method that automates the most laborious and subjective part of the serum neutralization assay – the determination of plaque number. Our results show good agreement between the visual and automated high throughput counting methods for determining RSV serum neutralization antibody titers.

These results were presented previously at the VI International Symposium on Respiratory Viral Infections, March 18–21, 2004, Fort Myers, Florida [5]

## Results

**Figure 1:**
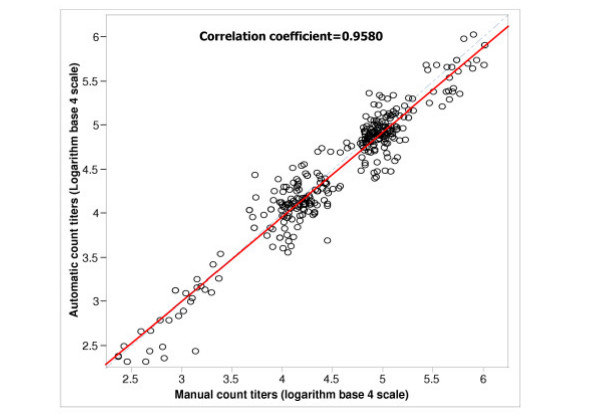
Scatter plot of automatically counted titers versus manually counted titers for each sample test presented in logarithm base 4 scale. The solid line is the simple linear regression line. The dotted line indicates the 45-degree line.

The data were analyzed by two general criteria: agreement and equivalence. Fig. 1. displays analysis of agreement between the two methods by plotting titers counted automatically against those counted manually. Pearson's correlation coefficient was 0.9580. Agreement between the titers obtained by the two methods was visualized by inspecting how closely the data spread around the 45-degree line (dashed line), which in this case, reflects the value of the correlation coefficient. The analysis indicated that there is a high level of agreement between the titers generated by image analysis and the standard plaque counting method.

**Figure 2:**
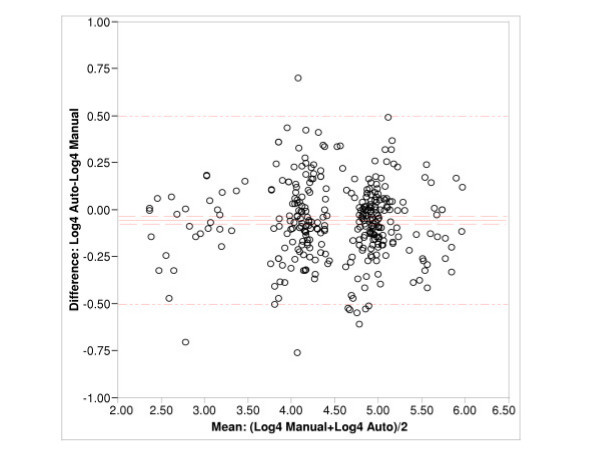
Difference-mean plot shows the difference of means in log 4 scale. The four populations of data grouped across the central line represent (from left to right) the low titer control serum, the reference serum tested without compliment, the reference serum tested with complement, and the high control serum group. The y-axis is represented in log4 scale the majority of the data lying within 0.5 log.

By plotting the difference between titer values of each data pair against the mean of the pair, we quantified the degree of equivalence for the majority of the data (Fig. 2). In log 4 scale, a measure of 1-log is equivalent to 1 dilution or a 4-fold change. The majority of the data lay within 0.5 log (or 2-fold) of the mean (Fig. 2) indicating that the two methods show equivalence within 2-fold.

**Figure 3:**
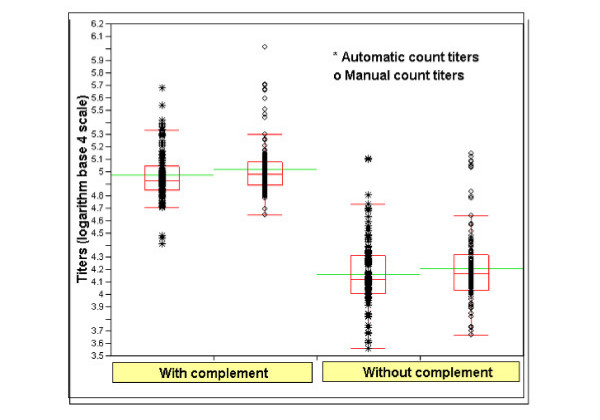
Boxplots of RSV reference serum titers with and without complement separated by counting methods. Open "o" represents manually counted titers and "*" represents automatically counted titers. For each group of data, the line drawn through the boxes represents the group mean; the top and bottom lines of the box represent the 25^th ^and 75^th ^percentiles; the brackets represent the 95^th ^percentile.

Additional analysis was performed on the largest subset of data to determine whether within assay variability would change when using the improved counting method. We evaluated all of the tests performed on the lyophilized reference serum in the presence and absence of complement and sorted the data by assay, method, and complement treatment. The range and mean of the replicate tests are depicted in Fig. 3. The difference in the mean of 136 replicates was less than 0.05 (log 4) between groups separated by complement treatment (Table 1). Table 1 indicated that the means and variability of the automatic and manual count were very similar.

**Table 1:** Summary of reference serum titers in logarithm base 4 scale.

**Complement**	**Counting Method**	**Mean Titer**	**Standard Deviation**	**Minimum**	**Maximum**	**Number**
With	Manual	5.02	0.215	4.65	6.01	136
	Automatic	4.97	0.204	4.41	5.69	136
Without	Manual	4.21	0.297	3.67	5.15	136
	Automatic	4.17	0.276	3.56	5.11	136

## Discussion

The data presented here demonstrate agreement and equivalence between traditional manual and automated plaque counting methods for detection of RSV neutralizing antibody titers. The 180 tests performed in the presence and absence of complement demonstrate that a wide range of titers is accurately detectable by both assays. The automated counting method does not increase the overall variability of the assay; rather variability was observed to be slightly lower with the automated method. Furthermore, we established that the two methods generated titers within 2-fold of each other. A clinically significant change of titer for an RSV patient is indicated by a 4-fold increase in titer, indicative of seroconversion [6]. Therefore, equivalence within 2-fold provides an acceptable level of confidence for the automated counting method.

The strength of this method is that it combines established plaque neutralization procedures with the technology of computerized image scanning and analysis. It has the advantage of providing more efficient and objective results while automating the most laborious and subjective aspect of the assay – plaque counting. Results can be stored as images and plaque counts indefinitely. This allows for better tracking of raw data, as is now mandated by federal and international regulatory bodies. Another important strength of this counting system is that it is capable of detecting and differentiating plaques of different morphology and thus can be used to assay many different viruses. In fact, we have already verified the capability of this system to read plaques created by mumps, influenza and other viruses (data not shown) whether in the context of determining viral potency or performing neutralization assays.

With the technological advances now available, the speed of plate scanning can be reduced further from 15 minutes/plate to approximately 2 minutes/plate. Robotic automation of plate loading can be introduced for further efficiency. The utilization of the TMB substrate staining also facilitates conservation of primary and secondary antibody stocks, which is an important factor when using viruses for which specific antibodies are not readily or commercially available. The objectivity and efficiency provided by this method of plaque counting facilitates the determination of RSV neutralizing antibody titers and can be readily applied to human epidemiology and vaccine clinical studies.

## Conclusion

In this report, we describe an RSV microneutralization assay that relies on automated plaque counting and provides a more rapid and less laborious method for detecting neutralizing antibodies to RSV. It provides equivalent results to the classical plaque neutralization assay and can be used in epidemiology and vaccine clinical studies.

## Materials and methods

### Serum samples

Human sera provided by Intergen Bio-Diagnostics (Purchase, NY) and Bioreclamation Inc. (Hicksville, NY), were tested for anti-RSV antibody titers. Sera were selected and pooled into 3 groups according to titer. The three groups consisted of a lyophilized reference serum, prepared under the auspices of NIAID and two control sera of high and low titer. An historical in-house control standard, C587645, was also tested. The reference serum made up the majority of tests (136 tests), whereas the control sera were each tested approximately 14 times. These sources of human sera comprised the specimens evaluated in this study and were collectively tested 180 times by both methods, in the presence and absence of complement.

### Virus and cells

The A2 strain of RSV was used as the challenge virus in all tests. Vero cells (ATCC Cat #CCL 34, ATCC, Rockville, MD) were cultured in EMEM with L-glutamine, 10% FBS, 1% of antibiotic/antimycotic, and non-essential amino acids. Cells were cultured on 96-well white opaque tissue culture plates (BD Falcon, Bedford, MA) for automated counting and on transparent 96-well plates (Corning-Costar, Corning, NY) for manual plaque counting 1–3 days prior to infection.

### Determination of RSV antibody titers

#### Microneutralization

Serum samples were heat-inactivated at 56°C for 30 minutes. Four-fold serial dilutions from 1:10 to 1:10,240 were prepared in virus diluent (EMEM with L-glutamine containing 2% FBS, 2.5% HEPES (1 M) and 1% antibiotic/antimycotic, 100×). All sera were tested in the presence and absence of 10% guinea pig complement (Cambrex/BioWhittaker, Walkersville, MD) which was added to the virus diluent prior to the addition of challenge virus. Serially diluted serum was challenged with an equal volume of the RSV-A2 strain, previously titered to give 50–100 pfu per 50 μl of inoculum. The serum/virus mixtures were incubated at 37°C, 5% CO_2 _for 1 h.

Vero cell monolayers, prepared in 96 well plates, were infected with 50 μl/well (in duplicate) of the serum/virus mixture. Plates were centrifuged at 1 h at 2000 rpm (700 g), followed by 30 min of rocking at room temperature. Supernatants were decanted. Plates were blotted and overlaid with 0.75% methyl cellulose (4,000 cP at 2% aqueous), prepared in MEM with 2% FBS, warmed to 37°C and inoculated at 100 μl/well. Plates were incubated at 37°C, 5% CO_2 _for 3 days to allow for plaque formation.

#### Conventional staining and plaque determination for RSV neutralization

Cells infected on transparent plates were fixed with a 50%:50% methanol:ethanol mixture at room temperature for 10 min. Plates were washed with DPBS after fixing and between staining steps. Plates were incubated for 1 h at 37°C, 5% CO_2 _with 50 μl/well of monoclonal antibody specific for RSV-A2 F-protein (Wyeth K6-5-1) diluted to 1:1,000 in Blotto (5% non-fat milk in PBS). Peroxidase labeled secondary goat anti-mouse IgG antibody (KPL, Gaithersburg, MD) diluted 1:100 in Blotto, was incubated at 50 μl/well for 1 h at room temperature. Plaques were developed using 100 μl/well 3,3'diaminobenzidine HRP substrate (0.5 mg/ml DAB, 0.01% H_2_O_2_) prepared in DPBS and incubated at room temperature for 5 – 10 minutes. Plates were washed with tap water to stop the reaction.

Plaques were counted manually by inverting the transparent plate under a dissecting microscope. The field of the well was separated into quadrants for ease of counting. Overlapping plaques were deemed individual when lobes were apparent.

#### TMB staining and plaque determination for RSV neutralization

Cells infected on opaque white tissue culture plates were fixed and washed as described above. Plates were incubated for 1 h at 37°C 5% CO_2 _with 50 μl/well of monoclonal antibody specific for RSV-A2 F-protein (Wyeth, K6-5-1) diluted to 1:10,000 in blotto. Peroxidase labeled secondary goat anti-mouse IgG antibody (KPL, Gaithersburg, MD) diluted 1:3,000 in Blotto was added at 50 μl/well and incubated for 1 hour at room temperature. Plaques were developed using 50 μl/well of a ready to use TMB precipitate HRP substrate, True Blue™ (KPL, Gaithersburg, MD). Higher dilutions of primary and secondary antibody were used due to the increased sensitivity of the peroxidase TMB substrate. Plates were washed thoroughly with tap water to stop the reaction and dried inverted in order to minimize bleaching.

**Figure 4:**
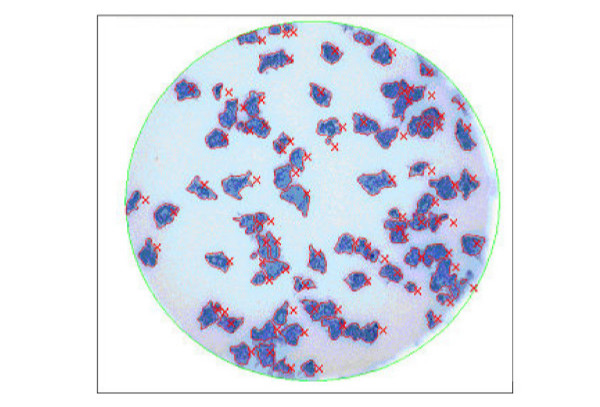
Vero cells infected with RSV-A2 virus and stained with True Blue™ peroxidase substrate. The image shows an example of plaque differentiation by automated counting. Each "x" represents one plaque counted by the image analyzer.

Plates were scanned and counted by the ImmunoSpot^® ^Image analyzer from Cellular Technology Ltd. (Cleveland, OH). The software, initially designed for use in ELISPOT analysis, has been successfully employed here for plaque detection and counting. Overlapping plaques were separated using a separation tolerance parameter set by the experimenter (Fig. 4). Minimum plaque size and sensitivity to stain comprised the major parameters that could be adjusted for counting. Parameters were adjusted on each experimental day, if necessary.

### Calculation of antibody titer

Titer was calculated from the average of duplicate sample wells by extrapolating the inverse dilution of serum that produced a 60% reduction of virus according to the following formula:

*X *= *(a-b)(e-c)/(c-d) *+ *a*

where, a = log_10 _of dilution above the 60% reduction point, b = log_10 _of dilution below the 60% reduction point, c = average plaque count above the 60% reduction point (corresponds with a), d = average plaque count below the 60% reduction point (corresponds with b) and e = value of 60% reduction of average virus control count.

### Statistical analysis

All titers were reported in logarithm base 4 scale in order to visually represent a difference of one dilution (of a 4-fold dilution series) as 1 log unit. Different statistical analyses were performed to assess the agreement of titers generated by two methods. In one analysis we graphically inspected the spread of the paired titers about the 45° line and computed Pearson's correlation coefficient. In another analysis, we determined the level of equivalence between the two assays, by constructing a difference-means plot [7].

## Competing interests

The author(s) declare that they have no competing interests.

## Authors' contributions

Design and conception of the study and co-drafted the manuscript (DPY); development of the methods and co-drafted the manuscript (EZ); assisted in the development of the automated plaque counting method (DL, HYW); statistical analysis of the data (JQ); manuscript preparation and review (RR). All authors read and approved the final manuscript.
